# Impacts of the US CDC recommendation on human papillomavirus vaccine uptake, 2010–2015

**DOI:** 10.3389/fpubh.2024.1464685

**Published:** 2024-12-18

**Authors:** Pallab K. Ghosh, Ahmed Chaudhry, Janis E. Campbell, Myongjin Kim, Kyle Smith, Firat Demir, Junying Zhao

**Affiliations:** ^1^Department of Economics, University of Oklahoma, Norman, OK, United States; ^2^Department of Biostatistics and Epidemiology, University of Oklahoma Health Sciences Center, Oklahoma, OK, United States; ^3^Department of Health Administration and Policy, University of Oklahoma Health Sciences Center, Oklahoma, OK, United States

**Keywords:** HPV, vaccination rate, vaccine uptake, gender, race, self-reported health status

## Abstract

**Objectives:**

As one type of vaccine policy, the effectiveness and spillover effects of the US CDC vaccine recommendations are inadequately evaluated. This study aims to fully evaluate its impacts on male adults, in addition to children, using better data.

**Design:**

A before-after study design to examine the CDC’s 2011 HPV vaccine recommendation for men aged 11–21.

**Data analysis:**

Individual-level data included the 2010–2015 US National Health Interview Survey full sample of 7,000 male children aged 11–18, younger adults aged 19–21 and 22–25, and older adults aged 26–60. Pooled cross-sectional surveys contained individual-level vaccination, socioeconomic, and demographic information. Outcome variable is an individual HPV vaccination status, measured as individual probability of HPV vaccination. Dummy regressions were estimated by a Linear Probability Model (LPM) with fixed effects for target and non-target age groups.

**Results:**

The policy was significantly associated with a 14.8% (*p* < 0.001) increased individual likelihood of HPV vaccination for men aged 11–21. It was also associated with a modest spillover effect, a 5.6% (*p* < 0.001) increased individual likelihood for men aged 22–25 and marginally for men aged 26–60. African American men and men with poor health were 2.7 and 15.4% less likely to uptake HPV vaccines than white men and men with good or fair health, respectively.

**Conclusion:**

This study complements the existing policy evaluation literature on HPV vaccine recommendation among male children by including adults and using better data. Findings offer comprehensive evidence of the effectiveness and spillover effects of this recommendation type of federal-level policy, provide policy lessons for other vaccines, and identify vulnerable subpopulations as targets for future policies.

## Background

Federal and state governments introduce vaccine policies. State-level policies typically include school immunization mandates, parent education mandates, and physician recommendations. Federal-level policies often encompass government subsidies and insurance cost reductions of vaccines [e.g., Affordable Care Act (ACA)], and consumer utilization recommendations by the Centers for Disease Control and Prevention (CDC) (1, 2). Existing literature evaluated the effectiveness of some policies in vaccination. Take the human papillomavirus (HPV) vaccine as an example. Churchill (2021) found that the ACA Medicaid expansion is associated with a 3–4% increase in the individual probability of a teenager initiating HPV vaccination (3). Moghtaderi and Adams found that state school immunization and parent education mandates are ineffective, while mandatory physician check-ups are effective in increasing HPV vaccination for adolescents aged 11–12 (4). In addition, physician recommendation had mixed results for adolescent females aged 11–17 based on cross-sectional survey data (5). However, existing studies regarding policy effectiveness in HPV population vaccination rate or individual vaccination likelihood have focused less on federal-level but state-level policies, and focused less on adults but a subset of adolescents aged 13–17. Moreover, state-level policies have limited impact compared to federal-level policies. Federal-level subsidy policies may increase consumer demand for vaccines nationwide but can be expensive for the government. For example, the ACA Medicaid expansion cost the US federal and state governments $628,819 million in fiscal year 2020 alone (6).

In contrast, from an economic perspective, federal-level vaccine recommendations directly influence consumer demand for vaccines and are inexpensive policies for the government, costing nearly $0 tax dollars. An official recommendation increases consumer demand for vaccines and incurs little monetary expenses to the government, whereas government subsidy or procurement of vaccines can cost millions of tax dollars a year. The CDC-Advisory Committee on Immunization Practices (ACIP) recommends who should get what vaccines and on what schedule. From an economic perspective, when there is a recommendation that tells a population that they should consume a vaccine, their quantity demanded for this vaccine thus final consumption would increase. Therefore, the CDC-ACIP recommendation is a consumption type of policy; it costs nearly $0 to the government and can work nationally. If it works, then it is better than other policies that are expensive and can only work regionally. Therefore, we ask the important question: Did the CDC recommendation work? However, its effectiveness in boosting vaccination has yet to be adequately evaluated in the existing literature.

In particular, since the US Food and Drug Administration licensed the Quadrivalent HPV (HPV4) vaccine for women in 2006 and expanded it to men in 2009 to prevent genital warts and anal cancer, there has been a concern about low uptake among men; the CDC-ACIP has made several HPV vaccine recommendations targeting subpopulations differing in gender, age, sexual orientation, and immunocompetence (7–9). In 2006, the CDC recommended the HPV vaccine for females aged 11–26. In 2009, the CDC-ACIP guided that the HPV vaccine may be given to males aged 9 through 26 years to reduce their likelihood of acquiring genital warts but did not recommend routine HPV vaccination for males (7). Thus, it was not yet a consumption policy for males. In 2011, the CDC-ACIP officially recommended three-dose routine HPV vaccination for males aged 11–12 and unvaccinated males aged 13–21 (8). However, in 2016, the CDC changed the 2011 policy to a two-dose series for males and females aged 9–14 but maintained the three-dose series for those aged 15–26. Yet, the 2011 CDC policy targeting men aged 11–21 was only evaluated on adolescents aged 13–15 or 17 by previous studies using the National Immunization Survey Teen (NIS-Teen) based on provider-reported vaccination histories; the NIS-Teen has over- and under-reporting issues and does not include targeted adolescent men at age 11–12 or adult men up to age 21 (10, 11).

Furthermore, what works to boost HPV vaccine uptake can be helpful policy lessons for other vaccines that have been witnessing low uptake in the US, a country facing unusual vaccine hesitancy challenges (12–15). In 2018, the US had an estimated HPV prevalence of 40.0% in men and women aged 15–59 years (16). In the US, each year, HPV causes about 37,800 of 47,984 new cases of cancer, 21,704 among men and 26,280 among women (17). During 2008–2016, 13,000 new cervical cancer cases were diagnosed in women annually and treated at a total cost of $56,250 per patient per year, totaling $731.3 million (18, 19). In 2020, 11,800 oropharyngeal cancers were diagnosed in men with total treatment costs of $139,749 per patient for the first two years, totaling $1.6 billion (20). The first HPV vaccine was approved for marketing in 2006 (21). It was a more recent vaccine and thus a better candidate for policy evaluation than other long-standing vaccines, such as polio and measles, which have experienced numerous policies and are challenging to differentiate the effect of each policy. The HPV vaccine is relatively expensive compared to other vaccines, costing $500–750 for three doses. Nevertheless, it is cost-beneficial in preventing infection and associated cancers, cheaper than cancer treatment and lifelong screening (22–25). However, the HPV vaccination rate among men remained low, less than 2% in 2010 (26). Interestingly, the rate among adolescent men successfully reached around 70% in 2020, toward the 80% Health People 2030 target (10, 27).

Therefore, to answer whether this inexpensive consumption-type policy works, we evaluate the impacts of the 2011 CDC recommendation on the HPV vaccination of adolescent and adult men aged 11–21. To complement the existing evaluation literature on this 2011 policy, we include all adolescents and adults targeted by this policy rather than a subset of adolescents, using US National Health Interview Survey (NHIS) data rather than NIS-Teen data. Although this policy only targeted men aged 11–21, it may motivate men outside this age group to also receive the vaccine. The increase in vaccination among non-targeted age groups is referred to as a spillover effect of the policy. Therefore, we use older age groups to investigate any positive spillover effects.

## Methods

### Data and study population

The US NHIS is conducted by the National Center for Health Statistics (NCHS) and is an annual cross-sectional household interview survey, targeting the civilian noninstitutionalized population who resides in all 50 states and the District of Columbia at the time of the interview. The annual dataset is obtained from the Integrated Public Use Microdata Series (IPUMS) Health Surveys: NHIS (28). The NHIS uses geographically clustered sampling techniques to select a nationally representative sample of dwelling units. Within each sampled household, one sample adult and one sample child are randomly selected. Thus, the NHIS sample is a nationally representative civilian noninstitutionalized population. We conduct a secondary data analysis using the NHIS pooled cross-sectional, rather than longitudinal, surveys that contain individual vaccination status. The surveys also contain individual demographics, self-reported health status, human capital, and household characteristics. Self-reported general health was rated on a five-point Likert scale in the NHIS, ranging from “excellent” to “poor.”

The CDC introduced the HPV vaccination policy for men aged 11–21 on October 25, 2011. Given the policy introduction in the fourth quarter of the year, the lack of monthly data, and delays in implementation and public awareness, we assume that the policy became effective in 2012. Moreover, we focus on the study period 2010–2015 because the NHIS has no data on male HPV vaccination before 2010, and the 2016 policy changed HPV vaccine dosage, making it harder to differentiate the effect of the 2011 policy. We restrict the sample to targeted men aged 11–21 but also examine non-targeted men aged 22–25, 26–40, and 41–60 for any spillover effects from the 2011 policy. Although the policy also made a recommendation to immunocompromised men aged 22–25, approximately only 4.7% of all-age men in the US have immunocompromised conditions (29). Thus, including or excluding this minimal proportion will not affect the main result for most men aged 22–25.

### Patient and public involvement

Patients and the public were not directly involved.

### Outcome variable

Our outcome variable is the HPV vaccination status of an individual. Vaccination status refers to the number of shots. For each individual, this outcome variable is calculated from the number of HPV shots received in response to the survey question. It is set equal to 1 if an individual takes at least one shot of the HPV vaccine and 0 otherwise. This variable has to be binary because the NHIS survey does not report the timing of the second and third shots; we restrict the sample to the first shot, thus one shot. We know that to be fully vaccinated during the study period 2010–2015 under the 2011 policy in effect, an individual needs three shots, with the second and third shots taken one to two and six months after the first shot, respectively (30). Thus, if an individual answered that the first shot was taken in a particular year, it is possible that the latter two shots were also in the same year; even if not, the non-adherence to the second and third shots does not affect our measure of at least one shot. The non-adherence to the latter two shots is an individual-level decision unrelated to when the CDC introduced the policy and thus again does not affect our evaluation of this policy.

This binary outcome variable is estimated by the Linear Probability Model (LPM); that is, it is an individual’s probability of HPV vaccination. Notice that an individual vaccination probability (a number between 0 and 1) is different from a population vaccination rate (%). In this study, the population vaccination rate is calculated as a proportion: Given an age group, the numerator is the number of individuals who received at least one dose, and the denominator is the number of all individuals in that age group. Therefore, the population vaccination rate represents the proportion of individuals who have received at least one dose, which is why we use “vaccination rate” and “coverage” interchangeably.

### Econometric analysis

V*_it_* is the HPV vaccination status for individual *i* and age group 11–21 in year *t*. Our main independent variable, *2011 HPV Policy Dummy*, takes the value of 1 for the years 2012 to 2015 and 0 otherwise to capture the effect of the CDC policy change. We estimate the following Eq. 1 using a LPM with fixed effects, employing STATA 17.0 statistical package:


(1) $$\begin{array}{c} V_{\mathrm{it}}=\alpha +\beta \times 2011\;HPV\;\mathrm{Policy Dummy}\\ +X\theta +\eta_{\mathrm{age}}+\delta_{\mathrm{region}-\mathrm{trend}}+\varepsilon_{\mathrm{it}} \end{array}$$

where *α* denotes an individual’s baseline vaccination probability without the policy. X denotes a set of control variables, including self-reported health status, family income, education, race and ethnicity. η*_age_* denotes the age-fixed effects that address any time-invariant unobserved heterogeneity for a specific age. The region-specific time trends, δ*_region-trend_*, address the regional time-variant unobserved factors that affect an individual’s vaccination. It includes four census regions of Northeast, Midwest, South, and West, and the Northeast is the default region.

We have used the LPM to estimate our coefficient of interest *β* because the advantages of using this model are that it is easy to interpret the coefficient, and it also produces closed-form estimates that can be computed directly from the data. However, the limitations of this simple model are that the predicted probabilities may lie outside 0 and 1, and the homoscedasticity assumption may not hold in the data. An alternative approach would be to use logistic regression, which is a non-linear model, and hence, it requires iterative procedures to estimate the model coefficients. To address the concern that our results depend on the LPM, we use the logistic regression models for a robustness check (see Appendix Tables S1–S3).

The *β* measures the impact of the policy change on the individual probability of HPV vaccination. Because we compare pre- and post-policy individual probabilities of vaccination for the same age group, the validity of our identification depends on the exogeneity of the timing of the policy. We argue that it is highly unlikely that the CDC recommendation was related to the pre-policy vaccination rate but was actually related to the new evidence of HPV vaccine efficacy for anal cancer. Moreover, there was no steep increase in men’s HPV infections right before the 2011 policy, which was supported by surveys of HPV prevalence during 2009–2010 (8, 31). Therefore, we treat the timing of the policy change as exogenous. Next, to address any concern about omitted variable bias, we include a list of control variables shown in Table 1 and three different fixed effects. Although we cannot control for all potential drivers of individual-level unobserved heterogeneity related to vaccine uptake, such as risk preference, social and family influence, adverse events due to an individual’s pre-existing health conditions, and time-varying individual characteristics, we include general self-reported health status and argue that time-varying individual characteristics are uncorrelated with the timing of the federal policy introduction. Hence, these individual-level unobserved variables do not raise a potential endogeneity concern. In addition, we conducted subgroup and sensitivity analyses using a two-way fixed effect model with age- and region-fixed effects instead of region-specific time trends. We used a similar method and data source for women for exploratory analysis.

**Table 1:** Summary statistics (mean and standard deviation) individual-level HPV vaccination status and other demographic characteristics for 2010–2015 for men aged 11–21, 15–21, and 18–21.

	Age 11–21			Age 15–21			Age 18–21		
	2010–2011	2012–2015	Diff	2010–2011	2012–2015	Diff	2010–2011	2012–2015	Diff
HPV vaccination status	2.0% (14.1)	11.6% (32.0)	9.6 [16.691]	1.2% (13.9)	11.6% (32.0)	10.4 [14.217]	2.1% (14.5)	11.6% (32.0)	9.5 [10.898]
Self-reported health status									
Excellent/Very Good	77.9% (−41.4)	78.7% (−40.9)	0.8 [0.808]	77.7% −41.7	78.7% −40.9	1.0 [0.913]	76.8% −42.2	78.6% −40.9	1.8 [1.384]
Good/Fair	21.6% (41.2)	21.0% (40.7)	−0.6 [−0.609]	21.9% (41.4)	21 0.0% (40.7)	−0.9 [−0.826]	22.8% (41.9)	22.8% (40.7)	0.0 [0.000]
Poor	0.4% (6.0)	0.3% (5.1)	−0.1 [−0.741]	0.4% (6.2)	0.3% (5.1)	−0.1 [−0.669]	0.3% (5.8)	0.3% (5.0)	0.0 [0.000]
Undefined	0.3% (1.6)	0.03% (1.8)	−0.27 [−6.635]	0.03% (1.9)	0.03% (1.8)	0.0 [0.0]	0.01% (2.6)	0.03% (1.8)	0.02 [0.304]
Education									
Grade 0–5	10.9% (31.2)	0.4% (6.2)	−10.5 [−18.520]	0.5% (6.8)	0.4% (6.2)	−0.1 [−0.581]	0.6% (7.7)	0.4% (6.2)	−0.2 [−0.946]
Grade 6–8	25.6% (43.6)	1.2% (10.2)	−24.4% [−0.149]	6.6% (24.8)	1.1% (10.2)	−5.5 [−11.302]	1.5% (12.1)	1.1% (10.2)	−0.4 [−1.172]
Grade 9-High School	45.7% (49.8)	50.6% (50.0)	4.9% [−4.085]	66.7% (47.1)	50.6% (50.0)	−16.1 [−12.454]	52.4% (49.9)	50.6% (50.0)	−1.8 [−1.145]
Some College	17.4% (37.9)	74.4% (49.9)	57 [54.284]	25.9% (43.8)	47.7% (49.9)	21.8 [17.391]	44.9% (49.8)	47.7% (49.9)	2.8 [1.784]
College and above	0.4% (6.1)	0.3% (5.1)	−0.1 [−0.733]	0.4% (6.2)	0.3% (5.1)	−0.1 [−0.669]	0.3% (5.2)	0.3% (5.1)	0.0 [0.000]
Family income									
Below 100 K	77.9% (41.5)	84.5% (36.2)	6.6 [6.998]	80.3% (39.7)	84.5% (36.2)	4.2 [4.182]	85.6% (35.1)	84.5% (36.2)	−1.1 [−0.975]
100 K and above	17.0% (37.5)	10.9% (31.1)	−6.1 [−7.292]	14.3% (35.0)	10.9% (31.1)	−3.4 [−3.890]	9.8% (29.7)	10.9% (31.1)	1.1 [1.140]
Undefined	5.1% (22.0)	4.7% (21.1)	−0.4 [−0.770]	5.4% (22.6)	4.7% (21.1)	−0.7 [−1.210]	4.6% (21.0)	4.7% (21.1)	0.1 [0.151]
Race									
White	72.3% (44.6)	73.6% (44.1)	1.3 [1.218]	73.2% (44.8)	73.6% (44.1)	0.4 [0.339]	73.1% (44.4)	73.6% (44.1)	0.5 [0.359]
Black	18.2% (38.6)	16.5% (37.2)	−1.7 [−1.861]	17.9% (38.4)	16.5% (37.2)	−1.4 [−1.397]	16.7% (37.4)	16.5% (37.2)	−0.2 [−0.171]
Others	9.5% (29.3)	9.9% (29.8)	0.4 [0.563]	9.8% (29.8)	9.9% (29.8)	0.1 [0.126]	10.2% (30.2)	9.8% (29.8)	−0.4 [−0.425]
Hispanic	28.4% (45.1)	25.2% (43.4)	−3.2 [−2.988]	27.8% (44.8)	25.2% (43.4)	−2.6 [−2.21]	27.9% (44.9)	25.2% (43.4)	−2.7 [−2.020]
No. of observations	3,874	3,126		2,602	3,126		1,493	3,126	

The numbers in the table represent the mean, and standard deviations are shown in the parenthesis. The third column of each panel shows the differences between 2012–2015 and 2010–2011 and t-statistics in square brackets. The Maximum and Minimum for each categorical variable are 1 and 0, respectively. Therefore, we represent the numbers in terms of percentage.

## Results

### Pre- and post-policy trends

**Figure 1:**
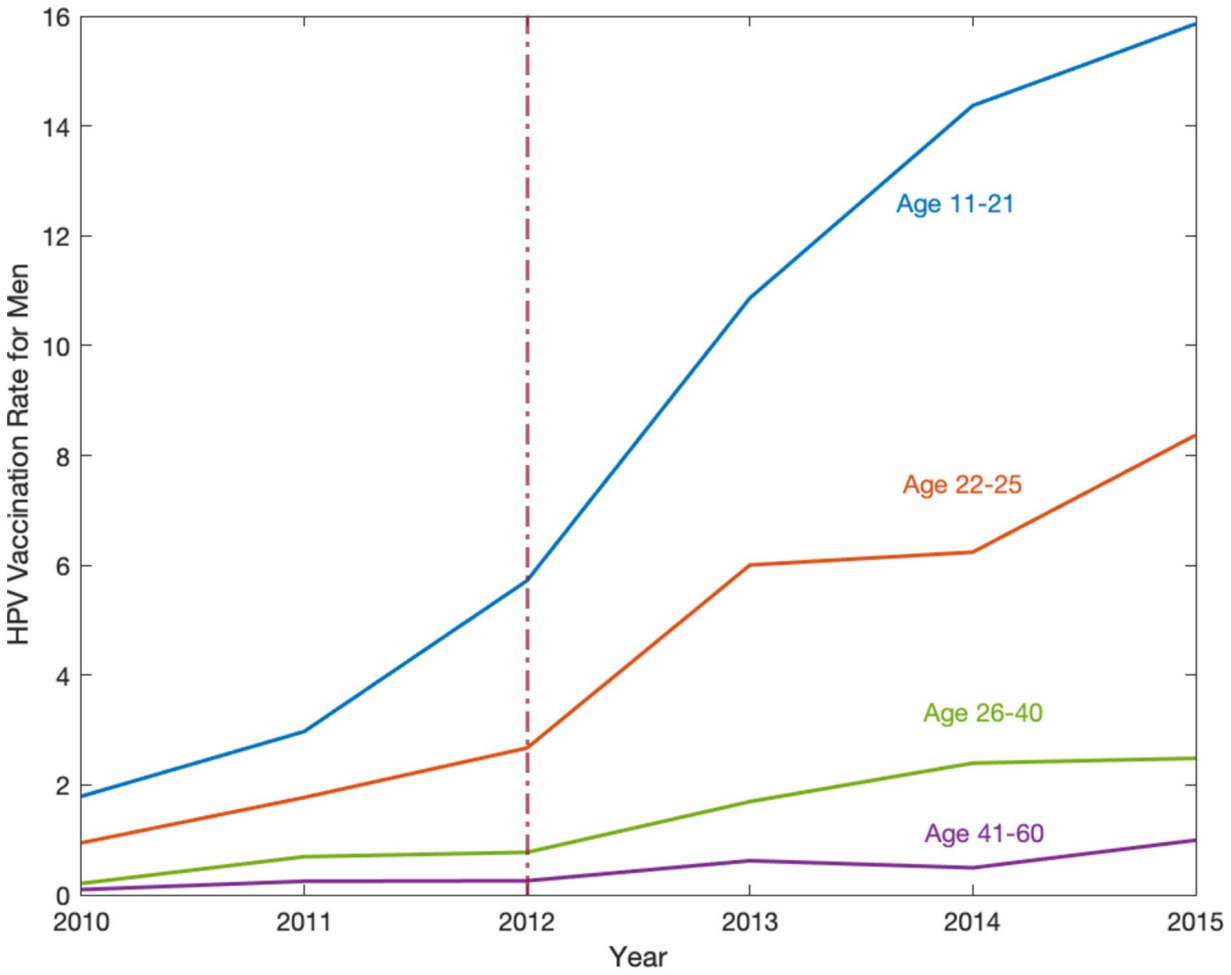
The HPV vaccination rate for men in the United States, 2010–2015. Source: Data are obtained from the National Health Interview Survey (NHIS) 2010–2015. The HPV vaccination rate implies how many individuals aged 11–60 take vaccines per 100 individuals. This figure shows the HPV vaccination rates for men aged 11–21, 22–25, 26–40, and 41–60. Trends are observed from 2010–2015 and analyzed before and after the recommendation policy, the red dashed line in 2012, to determine its effect on different age groups. After the implementation of the policy, the vaccination rate increased substantially for men aged 11–21 in the policy target group.

Figure 1 plots the HPV vaccination rates for men aged 11–21, 22–25, 26–40, and 41–60 to check the trends before and after the recommendation policy. After the implementation of the policy, the vaccination rate increased substantially for men aged 11–21 in the policy target group. We do not observe a similar jump in vaccination rates for men in other age groups.

### Descriptive results

Table 1 shows summary statistics (mean and standard deviation) of our sample of 7,000 individuals during 2010–2015. Using three panels for the male age groups, 11–21, 15–21, and 18–21, we show the variations in HPV population vaccination rate and other individual-level control variables for pre- and post-policy periods. The third column in each panel reports the difference between the post-policy period 2012–2015 and the pre-policy period 2010–2011. Notice that the population vaccination rate (%) in Table 1 summary statistics is not the individual vaccination probability (a number between 0 and 1) estimated by the LPM in Tables 2–6 and by the logistic model in Supplementary Tables S1–S3. Each categorical variable, such as self-reported health status, education, family income, and race, is represented by several dummy variables. These dummy variables are defined such that a value of 1 indicates the specific group of interest and 0 otherwise. Therefore, the mean of each dummy variable indicates its percentage point. We find that in the first panel for men aged 11–21, the population vaccination rate increased by 475%, from 2.0% in 2010–2011 to 11.6% in 2012–2015. Similarly in the second and third panels, compared to 2010–2011, population vaccination rates for men in two other policy target groups, aged 15–21 and 18–21, also significantly increased by 800% and 452.4% in 2012–2015, respectively. We also report the changes in all control variables in pre and post-policy periods and do not find any variables significantly changed after the policy, which is consistent with our intuition.

**Table 2:** Ordinary least squares estimates of the 2011 CDC recommendation policy effect on the HPV vaccination status for men aged 11–21.

	Dependent variable: vaccination status for men aged 11–21						
	(1)	(2)	(3)	(4)	(5)	(6)	(7)
HPV 2011 Policy Dummy	0.096*** (0.006)	0.097*** (0.007)	0.146*** (0.026)	0.147*** (0.026)	0.146*** (0.026)	0.149*** (0.026)	0.148*** (0.026)
Self-reported health status							
Good/Fair					0.005 (0.007)	0.008 (0.007)	0.008 (0.007)
Poor					−0.010 (0.048)	−0.007 (0.048)	−0.009 (0.048)
Undefined					−0.000 (0.010)	0.001 (0.021)	−0.000 (0.020)
Education							
Grade 6–8						−0.006 (0.014)	−0.006 (0.014)
Grade 9-High School						−0.017 (0.018)	−0.017 (0.018)
Some College						0.024 (0.019)	0.024 (0.019)
College and above						−0.047*** (0.018)	−0.042** (0.018)
Family income							
100 K and above							0.002 (0.008)
Undefined							−0.035*** (0.010)
Race							
African American				−0.009 (0.007)	−0.009 (0.008)	−0.006 (0.008)	−0.006 (0.008)
Other Races				−0.017* (0.010)	−0.017* (0.010)	−0.020** (0.010)	−0.019** (0.010)
Hispanic				−0.001 (0.007)	−0.002 (0.007)	0.002 (0.007)	0.002 (0.007)
Constant	0.020*** (0.002)	0.013** (0.007)	0.013 (0.008)	0.017* (0.009)	0.016* (0.009)	0.014 (0.009)	0.015 (0.010)
Age FEs	No	Yes	Yes	Yes	Yes	Yes	Yes
Region by Time Trend	No	No	Yes	Yes	Yes	Yes	Yes
No. of observations	7,000	7,000	7,000	7,000	7,000	7,000	7,000

FE is fixed effect. Robust standard errors are shown in parentheses. ***p < 0.01, **p < 0.05, *p < 0.1. The default (base group) for health status is excellent/very good, for education is Grade 0–5, for family income is below $100 K, for Race is White, and for Region is Northeast.

**Table 3:** The 2011 CDC recommendation policy effects on men’s HPV vaccination status by race and ethnicity, self-reported health status, education, and family income.

	Dependent variable: vaccination status for men aged 11–21				
	(1)	(2)	(3)	(4)	(5)
HPV 2011 Policy Dummy	0.156*** (0.026)	0.154*** (0.026)	0.148*** (0.026)	0.181* (0.100)	0.146*** (0.026)
By Race: base group: White					
HPV 2011 Policy Dummy *×* African American	−0.027* (0.016)				
HPV 2011 Policy Dummy *×* Other Races	−0.023 (0.020)				
By Hispanic status					
HPV 2011 Policy Dummy *×* Hispanic		−0.016 (0.014)			
Family income: Base Group: Below 100 K					
HPV 2011 Policy Dummy *×* 100 K and above			0.021 (0.021)		
HPV 2011 Policy Dummy *×* Undefined			−0.058** (0.023)		
By education: base group: Grade 0–5					
HPV 2011 Policy Dummy *×* Grade 6–8				−0.113 (0.101)	
HPV 2011 Policy Dummy *×* Grade 9-High School				−0.040 (0.097)	
HPV 2011 Policy Dummy *×* Some College				−0.022 (0.097)	
HPV 2011 Policy Dummy *×* College and above				−0.131 (0.097)	
By self-reported health status: base group: excellent/very good					
HPV 2011 Policy Dummy *×* Good/Fair					0.011 (0.015)
HPV 2011 Policy Dummy *×* Poor					−0.154** (0.075)
HPV 2011 Policy Dummy*Undefined					−0.051*** (0.018)
Self-reported health status					
Good/Fair	0.007 (0.007)	0.008 (0.007)	0.007 (0.007)	0.007 (0.007)	0.003 (0.006)
Poor	−0.008 (0.048)	−0.009 (0.048)	−0.009 (0.048)	−0.007 (0.048)	0.047 (0.069)
Undefined	−0.001 (0.023)	0.001 (0.017)	0.000 (0.020)	−0.005 (0.020)	0.025** (0.011)
Education					
Grade 6–8	−0.005 (0.014)	−0.006 (0.014)	−0.005 (0.014)	−0.001 (0.013)	−0.005 (0.014)
Grade 9-High School	−0.016 (0.018)	−0.018 (0.018)	−0.016 (0.017)	−0.022 (0.014)	−0.017 (0.018)
Some college	0.025 (0.019)	0.023 (0.019)	0.024 (0.019)	0.006 (0.016)	0.024 (0.019)
College and above	−0.042** (0.018)	−0.042** (0.018)	−0.046** (0.018)	−0.015 (0.014)	−0.042** (0.018)
Family income					
100 K and above	0.003 (0.008)	0.003 (0.008)	−0.005 (0.006)	0.002 (0.008)	0.002 (0.008)
Undefined	−0.035*** (0.010)	−0.035*** (0.010)	−0.011 (0.008)	−0.035*** (0.010)	−0.035*** (0.010)
Race					
African American	0.006 (0.007)	−0.006 (0.008)	−0.006 (0.008)	−0.006 (0.008)	−0.006 (0.008)
Other Races	−0.009 (0.007)	−0.019** (0.010)	−0.019** (0.010)	−0.019** (0.010)	−0.019* (0.010)
Hispanic	0.003 (0.007)	0.009 (0.006)	0.002 (0.007)	0.002 (0.007)	0.002 (0.007)
Constant	0.011 (0.009)	0.013 (0.010)	0.016* (0.009)	0.015 (0.009)	0.016* (0.010)
Age FE	Yes	Yes	Yes	Yes	Yes
Region by Time Trend	Yes	Yes	Yes	Yes	Yes
No. of observations	7,000	7,000	7,000	7,000	7,000

FE is fixed effect. Robust standard errors are shown in parentheses. ***p < 0.01, **p < 0.05, *p < 0.1. The default (base group) for health status is excellent/very good, for education is Grade 0–5, for family income is below $100 K, for Race is White, and for Region is Northeast.

**Table 4:** Spillover effects of the 2011 CDC recommendation policy for men aged 22–25, 26–40, and 41–60.

	Dependent variable: vaccination status for male age group 22–25					
	(1)	(2)	(3)	(4)	(5)	(6)
HPV 2011 Policy Dummy	0.045*** (0.005)	0.052*** (0.017)	0.053*** (0.017)	0.054*** (0.017)	0.057*** (0.017)	0.059*** (0.017)
Race: Base Group: White						
African American			0.003 (0.008)	0.003 (0.008)	0.007 (0.009)	0.007 (0.009)
Other Races			−0.000 (0.009)	−0.000 (0.009)	−0.004 (0.009)	−0.004 (0.009)
Hispanic			−0.008 (0.007)	−0.008 (0.007)	−0.002 (0.007)	−0.002 (0.007)
Self-reported health status						
Good/Fair				−0.001 (0.006)	0.003 (0.006)	0.003 (0.006)
Poor				0.010 (0.031)	0.019 (0.031)	0.019 (0.031)
Undefined				−0.009* (0.005)	−0.009 (0.006)	−0.009 (0.006)
Education						
Grade 6–8					0.050** (0.022)	0.051** (0.022)
Grade 9-High School					0.021*** (0.007)	0.022*** (0.007)
Some College					0.046*** (0.008)	0.046*** (0.008)
College and above					0.060*** (0.019)	0.061*** (0.019)
Family income						
100 K and above						−0.015 (0.010)
Undefined						−0.023** (0.011)
Constant	0.016*** (0.006)	0.010 (0.010)	0.011 (0.011)	0.011 (0.011)	−0.029** (0.013)	−0.029** (0.013)
Age FEs	No	Yes	Yes	Yes	Yes	Yes
Region by Time Trend	No	No	Yes	Yes	Yes	Yes
No. of observations	5,913	5,913	5,913	5,913	5,913	5,913

FE is fixed effect. Robust standard errors are shown in parentheses. ***p < 0.01, **p < 0.05, *p < 0.1. The default (base group) for health status is excellent/very good, for education is Grade 0–5, for family income is below $100 K, for Race is White, and for Region is Northeast.

**Table 5:** Spillover effects of the 2011 CDC recommendation policy for men aged 22–25, 26–40, and 41–60.

	Dependent variable: vaccination status for male age group 26–40					
	(1)	(2)	(3)	(4)	(5)	(6)
HPV 2011 Policy Dummy	0.013*** (0.001)	0.029*** (0.006)	0.030*** (0.006)	0.030*** (0.006)	0.030*** (0.006)	0.030*** (0.006)
Race						
African American			0.009*** (0.003)	0.009*** (0.003)	0.009*** (0.003)	0.010*** (0.003)
Other Races			0.001 (0.003)	0.001 (0.003)	0.000 (0.003)	0.000 (0.003)
Hispanic			−0.003* (0.002)	−0.003* (0.002)	−0.001 (0.002)	−0.001 (0.002)
Self-reported health status						
Good/Fair				0.003* (0.002)	0.004** (0.002)	0.004** (0.002)
Poor				0.031** (0.013)	0.033** (0.014)	0.033** (0.014)
Undefined				−0.009* (0.005)	−0.009 (0.006)	−0.009 (0.006)
Education						
Grade 6–8					0.002 (0.005)	0.002 (0.005)
Grade 9-High School					0.001 (0.004)	0.001 (0.004)
Some College					0.008* (0.005)	0.008* (0.005)
College and above					0.007 (0.005)	0.007 (0.005)
Family income						
100 K and above						0.001 (0.002)
Undefined						−0.001 (0.004)
Constant	0.014*** (0.004)	0.016*** (0.005)	0.015*** (0.005)	0.015*** (0.005)	0.008 (0.007)	0.008 (0.007)
Age FEs	No	Yes	Yes	Yes	Yes	Yes
Region by Time Trend	No	No	Yes	Yes	Yes	Yes
No. of observations	23,490	23,490	23,490	23,490	23,490	23,490

FE is fixed effect. Robust standard errors are shown in parentheses. ***p < 0.01, **p < 0.05, *p < 0.1. The default (base group) for health status is excellent/very good, for education is Grade 0–5, for family income is below $100 K, for Race is White, and for Region is Northeast.

**Table 6:** Spillover effects of the 2011 CDC recommendation policy for men aged 22–25, 26–40, and 41–60.

	Dependent variable: vaccination status for male age group 41–60					
	(1)	(2)	(3)	(4)	(5)	(6)
HPV 2011 Policy Dummy	0.004*** (0.001)	0.012*** (0.003)	0.012*** (0.003)	0.012*** (0.003)	0.012*** (0.003)	0.012*** (0.003)
Race						
African American			0.002* (0.001)	0.002 (0.001)	0.002* (0.001)	0.002* (0.001)
Other Races			0.001 (0.002)	0.001 (0.002)	0.001 (0.002)	0.001 (0.002)
Hispanic			−0.001 (0.001)	−0.001 (0.001)	−0.000 (0.001)	−0.000 (0.001)
Self-reported health status						
Good/Fair				0.000 (0.001)	0.001 (0.001)	0.001 (0.001)
Poor				0.001 (0.002)	0.002 (0.002)	0.002 (0.002)
Undefined				−0.006*** (0.002)	−0.006*** (0.002)	−0.006*** (0.002)
Education						
Grade 6–8					0.002 (0.001)	0.002 (0.001)
Grade 9-High School					0.003*** (0.001)	0.003*** (0.001)
Some College					0.006*** (0.001)	0.007*** (0.001)
College and above					0.006*** (0.002)	0.007*** (0.002)
Family income						
100 K and above						−0.001 (0.001)
Undefined						−0.003** (0.001)
Constant	0.002 (0.002)	−0.000 (0.002)	−0.000 (0.002)	−0.000 (0.002)	−0.006*** (0.002)	−0.005** (0.002)
Age FEs	No	Yes	Yes	Yes	Yes	Yes
Region by Time Trend	No	No	Yes	Yes	Yes	Yes
No. of observations	31,284	31,284	31,284	31,284	31,284	31,284

FE is fixed effect. Robust standard errors are shown in parentheses. ***p < 0.01, **p < 0.05, *p < 0.1. The default (base group) for health status is excellent/very good, for education is Grade 0–5, for family income is below $100 K, for Race is White, and for Region is Northeast.

### Main analysis results

Table 2 reports the LPM estimates of the policy effect on the HPV individual vaccination for men aged 11–21. In column (1), we do not include fixed effects and find a policy coefficient of 0.096 (*p* < 0.001), suggesting that the CDC recommendation increased 9.6% in the individual HPV vaccination probability to its pre-policy baseline probability among males aged 11–21. That is, an individual male in this age group is 9.6% more likely to get HPV vaccination after the policy. We add age-fixed effects in column (2), and region by time trends in column (3) to examine how much each explains the variation in vaccination. Moreover, in columns (4)–(7), we add four categorical control variables: self-reported health status, education, family income, and race and ethnicity. Overall, we find that the coefficient of the HPV policy variable remains stable at 0.148 (*p* < 0.001) in columns (3)–(7), indicating that the policy was associated with a 14.8% probability increase of HPV vaccination for an individual in the recommended age group 11–21.

Table 3 further reports the heterogeneous impacts of the policy by race and ethnicity, family income, education, and self-reported health status. In column (1), the coefficient of the interaction term between two variables, HPV policy and African American dummies, was −0.027 (*p* = 0.04), suggesting that within the policy target group aged 11–21, an African American male was 2.7% less likely to obtain HPV vaccination than a white male. We do not find such effects among any other racial or ethnic groups. Column (5), reporting the heterogeneous impact of self-reported health status, shows that a relatively less healthy individual was 15.4% (*p* = 0.06) less likely to take the HPV vaccine than an individual with good or fair health status.

### Sensitivity, subgroup, and exploratory analyses

Using a two-way fixed effect model with age- and region-fixed effects instead of region-specific time trends, sensitivity analysis results (Supplementary Tables S4–S6) are qualitatively the same as our main analysis results, validating our main estimation results.

In Figure 1, we notice a potential positive spillover effect on vaccination for the older male age group 22–25, but not for 26–40 or 41–60. Consistent with this observation, Table 4 shows that the policy had a modest, but still statistically and economically significant, spillover effect on young adult men aged 22–25, who were 5.6% (*p* < 0.001) more likely to take the HPV vaccine, even though the policy was recommended to the younger age group of 11–21. Similar to Table 2 results, we find that the coefficients of the policy dummy in columns (2)–(7) are stable (*p* < 0.001). Panels B and C show statistically significant 3.0% (*p* < 0.001) and 1.2% (*p* < 0.001) increases in the likelihood for older men aged 26–40 and 41–60, respectively. However, these increases were only one-fifth and one-twelfth of the substantial jump in vaccine uptake for men aged 11–21.

Moreover, Supplementary Figure S1 shows that the HPV population vaccination rate for women was approximately three times the rate for men. This may be related to the fact that the HPV vaccine has been recommended to women for a number of years earlier than men or that cervical cancer in women is more common than penile cancer in men and is more likely to be caused by HPV (32). Furthermore, the population vaccination rate for women aged 22–25 increased faster than that for younger women aged 11–21. This observation corresponds to the fact that while cervical cancer diagnosis before age 20 is rare; it increases from about 0 to 3 per 100,000 people from age 20–30 (33). These observations may also relate to a potential long-term effect of the earlier 2006 CDC recommendation targeting women aged 11–26. However, the outcome variable data in NHIS does not exist for women before 2008; therefore, we cannot test the latter hypothesis and call for future investigation.

## Discussion

We find that the 2011 CDC recommendation was associated with a 14.7% significant increase in the likelihood of getting vaccinated for men aged 11–21, a modest spillover effect of 5.6% for men aged 22–25, but little spillover effect for men in older age groups. Furthermore, African American men and men with poor health were significantly less likely to get the HPV vaccination. Our results fill in the literature gaps where the effects of federal vaccine recommendations on adult vaccination and spillover effects have not been studied. More importantly, from the economic and government perspectives, our study contributes to the literature that, unlike state-level and expensive vaccine policies, the CDC vaccine recommendation is a national-level inexpensive consumption policy that costs the federal government nearly $0 tax dollars; it effectively boosts individual likelihood of vaccination.

From economic and social perspectives, the recommendation policy also increases consumer welfare. First, suppose there exists a vaccine market without any policy; consumers buy vaccines in the market based on their ability to pay for the market price. In such a scenario, the vaccine recommendation policy introduces vaccines in an immunization schedule, thus essentially changing people’s vaccine consumption behavior by shifting the demand curve to the right and enlarging the consumer surplus. Therefore, the vaccine recommendation policy increases social welfare. Second, the routinely recommended vaccines are often paid for by the government so that consumers would face a lower price and, thus, higher demand for these recommended vaccines. By a similar reasoning, consumer welfare would further increase due to government subsidies for recommended vaccines.

Other economic perspectives are also relevant and helpful for understanding vaccination behaviors. First, herd immunity is conceptualized as a positive externality because herd immunity refers to indirect protection from an infectious disease that happens when a population is immune through vaccination or immunity developed through previous infection (34). Positive externality refers to benefits gained by individuals other than those who undertake an action and are not captured by a relevant market (35). In our setting, positive externality refers to indirect protection gained by unvaccinated or non-infected populations, aside from the vaccinated individuals, who are not consuming vaccines in the vaccine market.

Second, price elasticity of demand measures the responsiveness of consumer demand for a good (e.g., vaccine) to a change in its price. When the vaccine price increases, consumer demand for the vaccine decreases; the more demand decreases, the higher the price elasticity of demand. That is, such consumers are more sensitive to price (35). Literature showed that low-income consumers had relatively high price elasticities of demand for health care provided by nurses, community health workers, or traditional healers (36) and for primary goods, such as water (37) and food (38, 39). Future studies are needed on how vaccine prices affect demand. If low-income people are very sensitive to vaccine prices, they may receive government subsidies or other policy support for vaccines.

African American men’s relatively low likelihood of taking the HPV vaccine was similarly observed in their low uptake of other vaccines, such as COVID-19 and seasonal flu (40). This low uptake may be related to their continued distrust of the US health system and their financial barriers to vaccination due to lower income or insurance coverage than white males (41, 42). This low uptake is less likely related to the later recommendation to men than women because if it was, the low uptake should exist among men in all racial and ethnic groups rather than African American men only. On the other hand, males with poor health were relatively less likely than healthy males to get the HPV vaccine. This might be related to their health behaviors. General poor health may be a result of various bad health habits, such as smoking, drinking, and substance abuse, which show a relatively low demand for health, thus low demand for healthcare goods and services, such as HPV vaccinations. This may also be related to limited access to care. As of 2018, studies report that the majority (28 out of 50) of the US states have not allowed pharmacists to give HPV shots to individuals aged 11–12, and nine states have required a physician prescription (43). As a result, males who have less access to care, such as clinics, are more likely to have poor health and less likely to have HPV vaccinations. Therefore, other types of economic policies than vaccine recommendations, such as vaccination subsidies through insurance cost reduction or vaccination programs through government procurement, may target African American men and men with poor health statuses for which the vaccine recommendation was ineffective.

Our study has limitations stemming from the data. First, because the NHIS does not have the outcome variable for men until 2010, we had only two years of observations before the policy took effect. Also, the subsequent 2016 policy changed the dosage of a complete series from three to two for men aged 9–14, overlapping our sample aged 11–21 and limiting our study to four years between 2012 and 2015. Thus, we could not evaluate the long-term effects of the 2011 policy. However, NHIS is the only data collected by the CDC-NCHS that provides the outcome variable — the status of adult men’s HPV vaccination. Future policy evaluations require the NCHS to consider the usefulness of expected data in their survey design and the CDC-ACIP to consider the appropriate frequency and evaluability of their recommendations.

Second, without the NHIS geocodes, which were not available as of this writing, we could not examine state-level policies (e.g., school immunization mandates) and federal-state policy interactions with a difference-in-difference design, using individuals in states with mandates as the policy target group and individuals in states without mandates as the control group. We hope to carry out these tests once these requested geocodes are available and further evaluate other types of federal-level policies, such as the Vaccines for Children program that provides free HPV vaccines for uninsured, low-family income, and Native American children (44).

Finally, the NHIS data are self-reported and thus may have respondent biases such as social desirability bias. Nevertheless, the study has no missing data problem because NHIS surveys are pooled cross-sectional, rather than longitudinal, and thus do not follow a particular individual. The study has no misclassification problem because it compares the same group before and after the policy. There is no residual confounding as the NHIS is a well-established instrument and we conduct secondary, rather than primary, data analysis. The NHIS has a nationally representative sample of civilian noninstitutionalized populations but does not include military or institutionalized populations.

## Conclusion

The 2011 CDC HPV vaccine recommendation was associated with a substantial increase in vaccine uptake among adolescent and younger adult men, a modest or marginal increase among older men, but not among African American men or men with poor health. These findings provide evidence of the effectiveness of existing federal-level vaccine policies, offer policy lessons for other vaccines with low uptake, and identify vulnerable subpopulations as targets for other types of policies in the future.

## Data Availability

The original contributions presented in the study are included in the article/Supplementary material, further inquiries can be directed to the corresponding author.
