# Supplementary material for: Impacts of the US CDC recommendation on human papillomavirus vaccine uptake, 2010–2015
Source: Front Public Health. 2024 Dec 18;12:1464685. doi: 10.3389/fpubh.2024.1464685 (PMC11689657; doi:10.3389/fpubh.2024.1464685)
Supplement: Supplementary file 1 [file Data_Sheet_1.docx]

Supplementary Appendix (Online Not Intended for Publication): Impacts of the US CDC Recommendation on Human Papillomavirus Vaccine Uptake, 2010 - 2015

| **Figure S1: The trend of HPV vaccination rate for women in the United States, 2010-2015.** |
| --- |
| 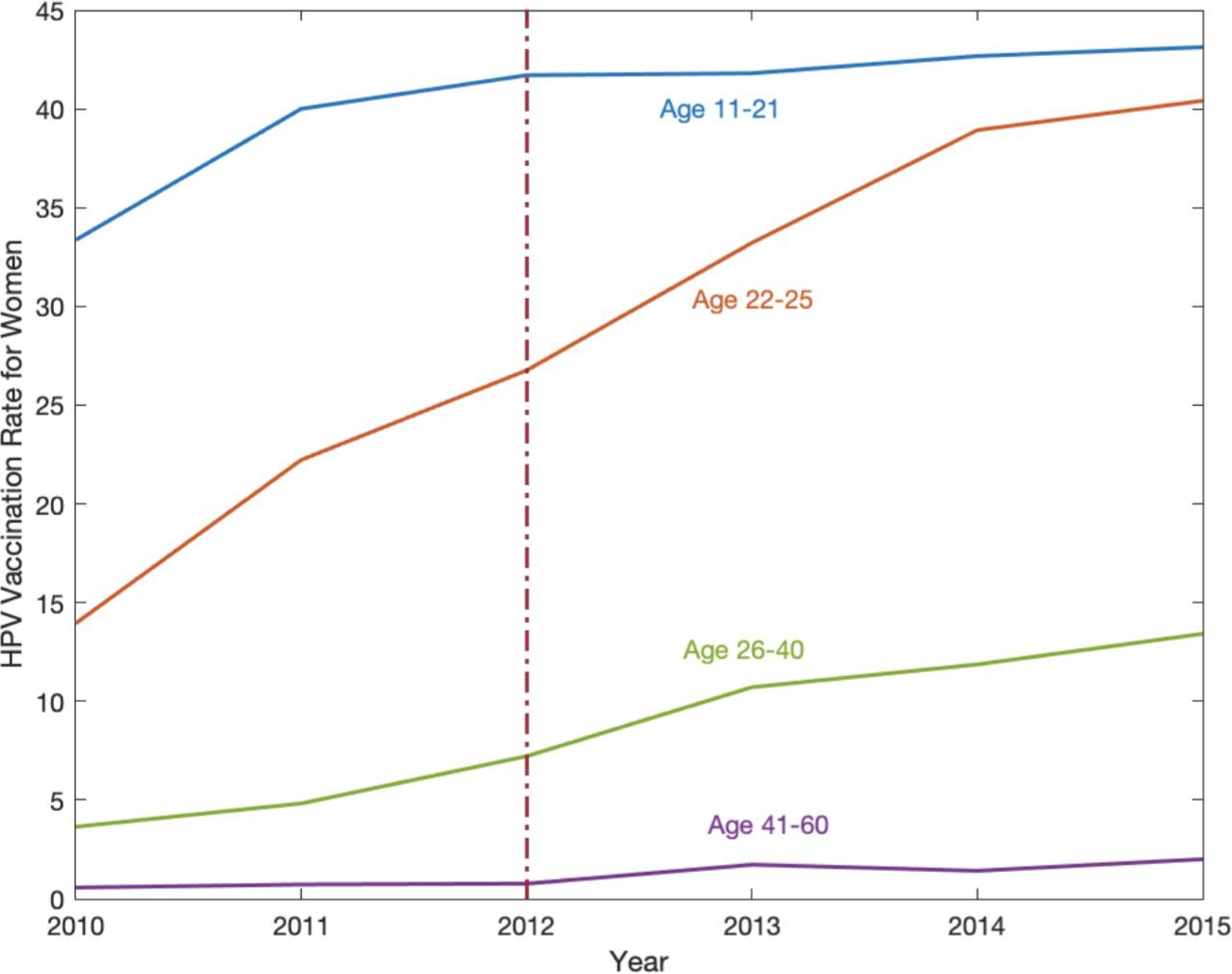 |
| Notes: The data are obtained from the National Health Interview Survey (NHIS) data 2010-2015. The HPV vaccination rate indicates how many individuals aged 11-60 take vaccines per 100 individuals. The figure shows the linear trend of annual HPV vaccination rates for women aged 11-21, 22-25, 26-40, and 41-60 before and after the HPV policy recommendation. The red dashed line in 2012 indicates the policy implementation year. The figure shows that after the implementation of the policy, the HPV vaccination rate increased substantially for women aged 11-21 and 22-25. |

| **Figure S2: The trend of HPV vaccination rate for men in the United States, 2010-2015.** |
| --- |
| 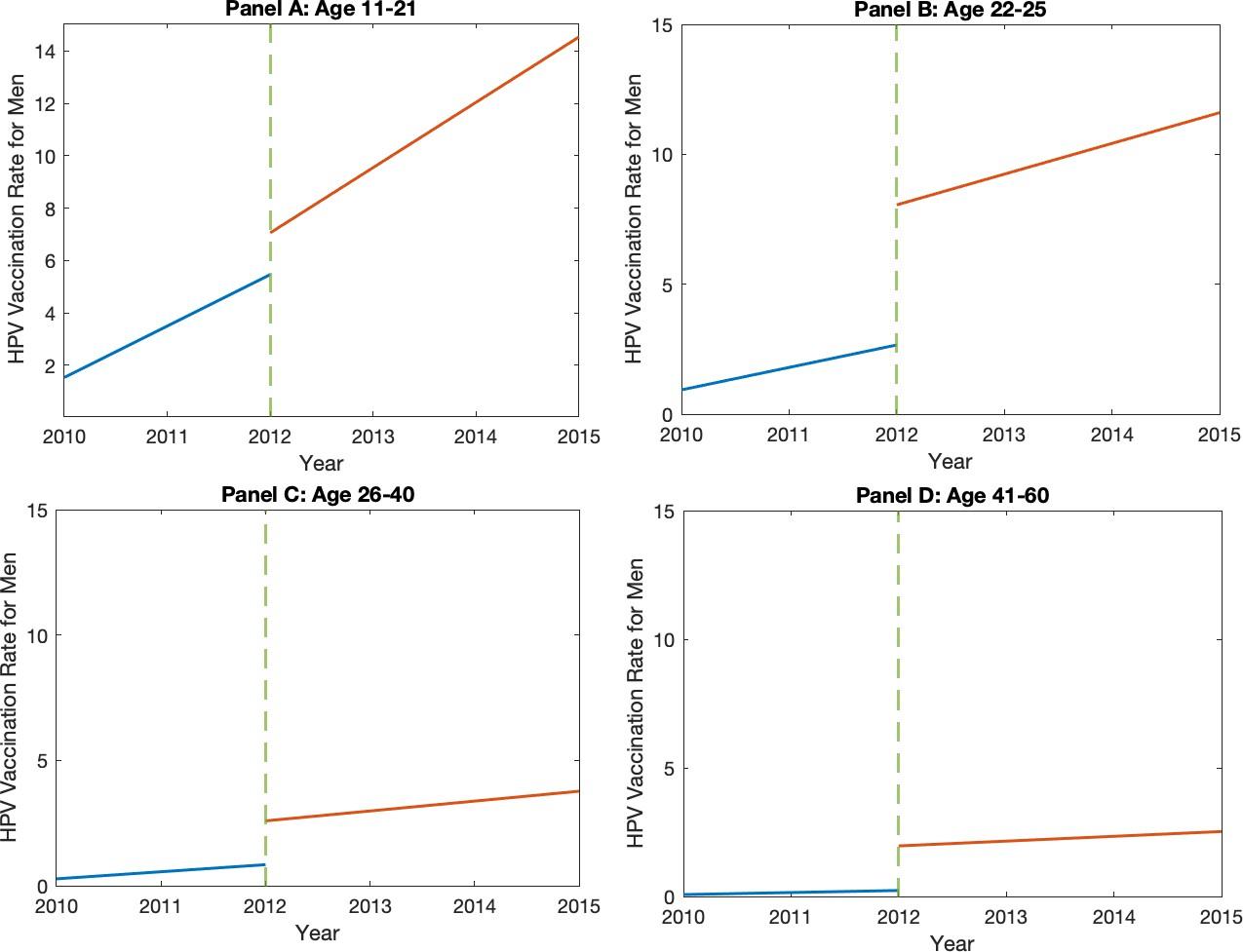 |
| Notes: The data are obtained from the National Health Interview Survey (NHIS) data 2010-2015. The HPV vaccination rate indicates how many individuals aged 11-60 take vaccines per 100 individuals. The figure shows the linear trend of annual HPV vaccination rates for men aged 11-21, 22-25, 26-40, and 41-60 before and after the HPV policy recommendation. The green dashed line in 2012 indicates the policy implementation year. The figure shows that after the implementation of the policy, the HPV vaccination rate increased substantially for men aged 11-21 (Panel A) and 22-25 (Panel B). |



| **Table S1: Logit model estimates of the 2011 CDC recommendation policy effect on the HPV vaccination status for men aged 11-21.** | | | | | | | | |
| --- | --- | --- | --- | --- | --- | --- | --- | --- |
|  | Dep Var: Vaccination Status for Men Aged 11-21 | | | | | | | |
|  | (1) | (2) | (3) | (4) |  | (5) | (6) | (7) |
| HPV 2011 Policy Dummy | 0.105*** | 0.103*** | 0.102*** | 0.102*** |  | 0.102*** | 0.102*** | 0.102*** |
| Self-Reported Health Status | (0.008) | (0.011) | (0.011) | (0.011) |  | (0.011) | (0.011) | (0.011) |
| Good/Fair |  |  |  |  |  | 0.006 | 0.009 | 0.008 |
|  |  |  |  |  |  | (0.007) | (0.007) | (0.007) |
| Poor |  |  |  |  |  | -0.016 | -0.015 | -0.016 |
| Undefined |  |  |  |  |  | (0.047) | (0.047) | (0.046) |
| Education |  |  |  |  |  |  |  |  |
| Grade 6-8 |  |  |  |  |  |  | -0.014 | -0.014 |
|  |  |  |  |  |  |  | (0.034) | (0.033) |
| Grade 9-High School |  |  |  |  |  |  | -0.031 | -0.031 |
|  |  |  |  |  |  |  | (0.034) | (0.033) |
| Some College |  |  |  |  |  |  | 0.002 | 0.002 |
| College and Above |  |  |  |  |  |  | (0.034) | (0.034) |
| Family Income |  |  |  |  |  |  |  |  |
| 100K and Above |  |  |  |  |  |  |  | 0.004 |
| Undefined Race |  |  |  |  |  |  |  | (0.009)  -0.030*** (0.010) |
| Black |  |  |  | -0.010 |  | -0.011 | -0.008 | -0.008 |
|  |  |  |  | (0.008) |  | (0.008) | (0.008) | (0.008) |
| Other Races |  |  |  | -0.014 |  | -0.014 | -0.017** | -0.016* |
| Hispanic |  |  |  | (0.009) |  | (0.009) | (0.009) | (0.009) |
|  |  |  |  | -0.001 |  | -0.001 | 0.001 | 0.002 |
| Region |  |  |  | (0.007) |  | (0.007) | (0.007) | (0.007) |
| North Central/Midwest |  |  | -0.026*** | -0.027*** |  | -0.027*** | -0.029*** | -0.030*** |
| South West |  |  | (0.010)  -0.035*** (0.009)  -0.018* (0.010) | (0.010)  -0.035*** (0.009)  -0.018* (0.010) |  | (0.010)  -0.035*** (0.009)  -0.018* (0.010) | (0.010)  -0.036*** (0.009)  -0.019* (0.010) | (0.010)  -0.036*** (0.009)  -0.019* (0.010) |
| Age FEs | No | Yes | Yes | Yes |  | Yes | Yes | Yes |
| Regions by Time Trend | No | No | Yes | Yes |  | Yes | Yes | Yes |
| No of Observations | 7,000 | 7,000 | 7,000 | 7,000 |  | 6,998 | 6,973 | 6,973 |
| Notes: FE is fixed effect. Robust standard errors are shown in parentheses. *** p*<*0·01, ** p*<*0·05, * p*<*0·1. The default (base group) for Health Status is Excellent/Very good, for Education is Grade 0-5, for Family Income is Below $100K, for Race is White, and for Region is Northeast. | | | | | | | | |

| **Table S2: The 2011 CDC recommendation policy effects on men’s HPV vaccination status by race and ethnicity, self-reported health status, education, and family income.** | | | | |
| --- | --- | --- | --- | --- |
|  | Dep Var: Vaccination Status for Men Aged 11-21 | | | |
|  | (1) | (2) | (3) | (4) |
| HPV 2011 Policy Dummy | 0.094*** | 0.094*** | 0.095*** | 0.076*** |
| By Race | (0.011) | (0.011) | (0.011) | (0.009) |
| HPV 2011 Policy Dummy *×* Black | -0.510 |  |  |  |
|  | (0.329) |  |  |  |
| HPV 2011 Policy Dummy *×* Other Races | 0.214 |  |  |  |
| By Hispanic Status | (0.512) |  |  |  |
| HPV 2011 Policy Dummy *×* Hispanic |  | -0.377 |  |  |
| Family Income |  | (0.273) |  |  |
| HPV 2011 Policy Dummy *×* 100K and Above |  |  | 0.310 |  |
|  |  |  | (0.352) |  |
| HPV 2011 Policy Dummy *×* Undefined |  |  | 0.087 |  |
| By Education |  |  | (0.802) |  |
| HPV 2011 Policy Dummy *×* Grade 6-8 |  |  |  | -1.713 |
|  |  |  |  | (1.406) |
| HPV 2011 Policy Dummy *×* Grade 9-High School |  |  |  | 0.474 |
|  |  |  |  | (0.937) |
| HPV 2011 Policy Dummy *×* Some College |  |  |  | 0.474 |
| HPV 2011 Policy Dummy *×* College and Above |  |  |  | (0.965) |
| Self-Reported Health Status |  |  |  |  |
| Good/Fair | 0.008 | 0.008 | 0.008 | 0.008 |
|  | (0.007) | (0.007) | (0.007) | (0.007) |
| Poor | -0.018 | -0.017 | -0.017 | -0.015 |
| Undefined | (0.045) | (0.046) | (0.046) | (0.047) |
| Education |  |  |  |  |
| Grade 6-8 | -0.013 | -0.014 | -0.013 | -0.057 |
|  | (0.033) | (0.033) | (0.033) | (0.045) |
| Grade 9-High School | -0.030 | -0.032 | -0.030 | -0.044 |
|  | (0.033) | (0.033) | (0.033) | (0.043) |
| Some College | 0.003 | 0.001 | 0.003 | -0.013 |
| College and Above | (0.033) | (0.034) | (0.034) | (0.043) |
| Race |  |  |  |  |
| Black | -0.009 | -0.008 | -0.008 | -0.008 |
| Other Races  Hispanic | (0.008)  -0.016*  (0.009) | (0.008)  -0.016*  (0.009) | (0.008)  -0.017*  (0.009) | (0.008)  -0.016*  (0.009) |
|  | 0.002 | 0.001 | 0.002 | 0.002 |
|  | (0.007) | (0.007) | (0.007) | (0.007) |
| Other Controls | Yes | Yes | Yes | Yes |
| Age FE | Yes | Yes | Yes | Yes |
| No of Observations | 6,973 | 6,973 | 6,973 | 6,973 |
| Notes: FE is fixed effect. Robust standard errors are shown in parentheses. *** p*<*0·01, ** p*<*0·05, * p*<*0·1. The default (base group) for Health Status is Excellent/Very good, for Education is Grade 0-5, for Family Income is Below $100K, for Race is White, and for Region is Northeast. Other controls include family income. | | | | |

| **Table S3: (Panel A) Spillover effects of the 2011 CDC recommendation policy for men aged 22-25, 26-40, and 41-60.** | | | | | | |
| --- | --- | --- | --- | --- | --- | --- |
|  | Dep Var: Vaccination Status for Men Aged 22-25 | | | | | |
|  | (1) | (2) | (3) | (4) | (5) | (6) |
| HPV 2011 Policy Dummy | 0.062*** | 0.062*** | 0.062*** | 0.062*** | 0.062*** | 0.062*** |
| Race | (0.009) | (0.009) | (0.009) | (0.009) | (0.009) | (0.009) |
| Black |  |  | 0.003 | 0.003 | 0.008 | 0.008 |
|  |  |  | (0.009) | (0.009) | (0.009) | (0.009) |
| Other Races |  |  | -0.001 | -0.001 | -0.005 | -0.005 |
| Hispanic |  |  | (0.008) | (0.008) | (0.008) | (0.008) |
|  |  |  | -0.009 | -0.009 | -0.003 | -0.003 |
| Self-Reported Health Status |  |  | (0.006) | (0.006) | (0.007) | (0.007) |
| Good/Fair |  |  |  | -0.002 | 0.003 | 0.003 |
|  |  |  |  | (0.006) | (0.007) | (0.007) |
| Poor |  |  |  | 0.010 | 0.025 | 0.025 |
| Undefined |  |  |  | (0.038) | (0.047) | (0.047) |
| Family Income |  |  |  |  |  |  |
| 100K and Above |  |  |  |  |  | -0.012 |
| Undefined Region |  |  |  |  |  | (0.009)  -0.020* (0.010) |
| North Central/Midwest |  | -0.018* | -0.019* | -0.019* | -0.018* | -0.020** |
| South |  | (0.010)  -0.017* (0.009) | (0.010)  -0.017* (0.009) | (0.010)  -0.017* (0.009) | (0.010)  -0.018* (0.009) | (0.010)  -0.019** (0.010) |
| West |  | -0.013 | -0.012 | -0.012 | -0.011 | -0.013 |
|  |  | (0.010) | (0.010) | (0.010) | (0.010) | (0.010) |
| Constant | -4.215*** | -3.918*** | -3.880*** | -3.873*** | -3.407*** | -3.333*** |
|  | (0.231) | (0.281) | (0.288) | (0.290) | (0.418) | (0.418) |
| Age FEs | No | Yes | Yes | Yes | Yes | Yes |
| No of Observations | 5,913 | 5,913 | 5,913 | 5,913 | 5,865 | 5,865 |
| Notes: FE is fixed effect. Robust standard errors are shown in parentheses. *** p*<*0·01, ** p*<*0·05, * p*<*0·1. The default (base group) for Health Status is Excellent/Very good, for Education is Grade 0-5, for Family Income is Below $100K, for Race is White, and for Region is Northeast. Other controls include family income. | | | | | | |

| **Table S3: (Panel B) Spillover effects of the 2011 CDC recommendation policy for men aged 22-25, 26-40, and 41-60.** | | | | | | |
| --- | --- | --- | --- | --- | --- | --- |
|  | Dep Var: Vaccination Status for Men Aged 26-40 | | | | | |
|  | (1) | (2) | (3) | (4) | (5) | (6) |
| HPV 2011 Policy Dummy | 0.019*** | 0.019*** | 0.019*** | 0.019*** | 0.019*** | 0.019*** |
| Race | (0.003) | (0.003) | (0.003) | (0.003) | (0.003) | (0.003) |
| Black |  |  | 0.009*** | 0.009*** | 0.010*** | 0.010*** |
|  |  |  | (0.003) | (0.003) | (0.003) | (0.003) |
| Other Races |  |  | 0.000 | 0.000 | -0.000 | -0.000 |
| Hispanic |  |  | (0.002) | (0.002) | (0.002) | (0.002) |
|  |  |  | -0.004** | -0.004** | -0.002 | -0.001 |
| Self-Reported Health Status |  |  | (0.002) | (0.002) | (0.002) | (0.002) |
| Good/Fair Poor  Undefined |  |  |  | 0.003* (0.002) 0.032** (0.015) | 0.004**  (0.002)  0.040**  (0.017) | 0.004**  (0.002)  0.041**  (0.017) |
| Education |  |  |  |  |  |  |
| Grade 6-8 |  |  |  |  | 0.002 | 0.002 |
|  |  |  |  |  | (0.006) | (0.006) |
| Grade 9-High School |  |  |  |  | 0.003 | 0.003 |
| Some College |  |  |  |  | (0.005)  0.010*  (0.005) | (0.005)  0.010*  (0.005) |
| College and Above |  |  |  |  | 0.009 | 0.009 |
| Family Income |  |  |  |  | (0.006) | (0.006) |
| 100K and Above |  |  |  |  |  | 0.002 |
|  |  |  |  |  |  | (0.002) |
| Undefined |  |  |  |  |  | 0.000 |
| Region |  |  |  |  |  | (0.004) |
| North Central/Midwest |  | -0.007** | -0.006** | -0.007** | -0.007** | -0.006** |
| South |  | (0.003)  -0.005* (0.003) | (0.003)  -0.005** (0.002) | (0.003)  -0.005**  (0.003) | (0.003)  -0.005**  (0.003) | (0.003)  -0.005**  (0.003) |
| West |  | 0.002 | 0.004 | 0.003 | 0.003 | 0.003 |
|  |  | (0.003) | (0.003) | (0.003) | (0.003) | (0.003) |
| Age FEs | No | Yes | Yes | Yes | Yes | Yes |
| No of Observations | 23,490 | 23,490 | 23,490 | 23,484 | 23,484 | 23,484 |
| Notes: FE is fixed effect. Robust standard errors are shown in parentheses. *** p*<*0·01, ** p*<*0·05, * p*<*0·1. The default (base group) for Health Status is Excellent/Very good, for Education is Grade 0-5, for Family Income is Below $100K, for Race is White, and for Region is Northeast. Other controls include family income. | | | | | | |



| **Table S3: (Panel C) Spillover effects of the 2011 CDC recommendation policy for men aged 22-25, 26-40, and 41-60.** | | | | | | |
| --- | --- | --- | --- | --- | --- | --- |
|  | Dep Var: Vaccination Status for Men Aged 21-60 | | | | | |
|  | (1) | (2) | (3) | (4) | (5) | (6) |
| HPV 2011 Policy Dummy | 0.005*** | 0.005*** | 0.005*** | 0.005*** | 0.006*** | 0.006*** |
| Race | (0.001) | (0.001) | (0.001) | (0.001) | (0.001) | (0.001) |
| Black |  |  | 0.002 | 0.002 | 0.003* | 0.002 |
|  |  |  | (0.001) | (0.001) | (0.001) | (0.001) |
| Other Races |  |  | 0.001 | 0.001 | 0.001 | 0.001 |
| Hispanic |  |  | (0.002) | (0.002) | (0.001) | (0.002) |
|  |  |  | -0.001 | -0.001 | 0.000 | -0.000 |
| Self-Reported Health Status |  |  | (0.001) | (0.001) | (0.001) | (0.001) |
| Good/Fair |  |  |  | 0.000 | 0.001 | 0.001 |
|  |  |  |  | (0.001) | (0.001) | (0.001) |
| Poor |  |  |  | 0.001 | 0.002 | 0.002 |
| Undefined |  |  |  | (0.002) | (0.003) | (0.003) |
| Education |  |  |  |  |  |  |
| Grade 6-8 |  |  |  |  |  |  |
| Grade 9-High School |  |  |  |  |  |  |
| Some College |  |  |  |  |  |  |
| College and Above |  |  |  |  |  |  |
| Family Income |  |  |  |  |  |  |
| 100K and Above |  |  |  |  |  | -0.001 |
| Undefined Region |  |  |  |  |  | (0.001)  -0.003** (0.001) |
| North Central/Midwest |  | 0.000 | 0.000 | 0.000 | 0.000 | -0.000 |
|  |  | (0.001) | (0.001) | (0.001) | (0.001) | (0.001) |
| South |  | 0.002* | 0.002 | 0.002 | 0.002 | 0.002 |
| West |  | (0.001)  0.003** (0.001) | (0.001)  0.003** (0.001) | (0.001)  0.003** (0.001) | (0.001)  0.003** (0.001) | (0.001)  0.003** (0.001) |
| Age FEs | No | Yes | Yes | Yes | Yes | Yes |
| No of Observations | 31,284 | 31,284 | 31,284 | 31,272 | 30,682 | 30,682 |
| Notes: FE is fixed effect. Robust standard errors are shown in parentheses. *** p*<*0·01, ** p*<*0·05, * p*<*0·1. The default (base group) for Health Status is Excellent/Very good, for Education is Grade 0-5, for Family Income is Below $100K, for Race is White, and for Region is Northeast. Other controls include family income. | | | | | | |

| **Table S4: Ordinary least squares estimates of the 2011 CDC recommendation policy effect on the HPV vaccination status for men aged 11-21, without region by time trend.** | | | | | | | |
| --- | --- | --- | --- | --- | --- | --- | --- |
|  | Dependent Variable: Vaccination Status for Men Aged 11-21 | | | | | | |
|  | (1) | (2) | (3) | (4) | (5) | (6) | (7) |
| HPV 2011 Policy | 0·096*** | 0·097*** | 0·097*** | 0·096*** | 0·097*** | 0·096*** | 0·096*** |
| Dummy | (0·006) | (0·007) | (0·007) | (0·007) | (0·007) | (0·007) | (0·007) |
|  |  |  |  |  |  |  |  |
| Self-Reported Health Status |  |  |  |  |  |  |  |
| Good/Fair |  |  |  |  | 0·007 | 0·010 | 0·010 |
|  |  |  |  |  | (0·007) | (0·007) | (0·007) |
| Poor |  |  |  |  | -0·019 | -0·016 | -0·018 |
|  |  |  |  |  | (0·048) | (0·048) | (0·048) |
| Undefined |  |  |  |  | -0·049*** | -0·048 | -0·049 |
|  |  |  |  |  | (0·018) | (0·031) | (0·031) |
| Education |  |  |  |  |  |  |  |
| Grade 6-8 |  |  |  |  |  | -0·009 | -0·009 |
|  |  |  |  |  |  | (0·014) | (0·014) |
| Grade 9-High School |  |  |  |  |  | -0·022 | -0·022 |
|  |  |  |  |  |  | (0·018) | (0·018) |
| Some College |  |  |  |  |  | 0·018 | 0·018 |
|  |  |  |  |  |  | (0·020) | (0·020) |
| College and Above |  |  |  |  |  | -0·050*** | -0·046** |
|  |  |  |  |  |  | (0·018) | (0·019) |
| Family Income |  |  |  |  |  |  |  |
| 100K and Above |  |  |  |  |  |  | 0·003 |
|  |  |  |  |  |  |  | (0·009) |
| Undefined |  |  |  |  |  |  | -0·030*** |
|  |  |  |  |  |  |  | (0·010) |
| Race |  |  |  |  |  |  |  |
| Black |  |  |  | -0·011 | -0·011 | -0·008 | -0·008 |
|  |  |  |  | (0·007) | (0·008) | (0·008) | (0·008) |
| Other Races |  |  |  | -0·014 | -0·015 | -0·018* | -0·017* |
|  |  |  |  | (0·010) | (0·010) | (0·010) | (0·010) |
| Hispanic |  |  |  |  |  |  |  |
|  |  |  |  | -0·001 | -0·002 | 0·001 | 0·002 |
|  |  |  |  | (0·007) | (0·007) | (0·007) | (0·007) |
| Region |  |  |  |  |  |  |  |
| North |  |  | -0·026*** | -0·028*** | -0·028*** | -0·029*** | -0·029*** |
| Central/Midwest |  |  | (0·010) | (0·010) | (0·010) | (0·010) | (0·010) |
|  |  |  |  |  |  |  |  |
| South |  |  | -0·036*** | -0·036*** | -0·036*** | -0·036*** | -0·036*** |
|  |  |  | (0·009) | (0·009) | (0·009) | (0·009) | (0·009) |
| West |  |  | -0·018* | -0·019* | -0·019* | -0·019* | -0·019* |
|  |  |  | (0·010) | (0·010) | (0·010) | (0·010) | (0·010) |
| Constant | 0·020*** | 0·013** | 0·038*** | 0·042*** | 0·041*** | 0·040*** | 0·041*** |
|  | (0·002) | (0·007) | (0·010) | (0·011) | (0·011) | (0·011) | (0·011) |
|  |  |  |  |  |  |  |  |
| Age FEs | No | Yes | Yes | Yes | Yes | Yes | Yes |
| No of Observations | 7,000 | 7,000 | 7,000 | 7,000 | 7,000 | 7,000 | 7,000 |
| Notes: FE is fixed effect. Robust standard errors are shown in parentheses. *** p<0·01, ** p<0·05, * p<0·1. The default (base-group) for Health Status is Excellent/Very good, for Education is Grade 0-5, for Family Income is Below $100K, for Race is White, and for Region is Northeast. | | | | | | | |

| **Table S5: The 2011 CDC recommendation policy effects on men’s HPV vaccination status by race and ethnicity, self-reported health status, education, and family income, without region by time trend.** | | | | | |
| --- | --- | --- | --- | --- | --- |
|  | Dependent Variable: Vaccination Status for Men Aged 11-21 | | | | |
|  | (1) | (2) | (3) | (4) | (5) |
| HPV 2011 Policy Dummy | 0·103*** | 0·098*** | 0·095*** | 0·131 | 0·093*** |
|  | (0·008) | (0·008) | (0·007) | (0·103) | (0·008) |
| By Race: Base Group: White |  |  |  |  |  |
| HPV 2011 Policy Dummy *×* Black | -0·034** |  |  |  |  |
|  | (0·016) |  |  |  |  |
| HPV 2011 Policy Dummy *×* Other Races | -0·010 |  |  |  |  |
|  | (0·020) |  |  |  |  |
| By Hispanic Status |  |  |  |  |  |
| HPV 2011 Policy Dummy *×* Hispanic |  | -0·009 |  |  |  |
|  |  | (0·014) |  |  |  |
| Family Income: Base Group: Below 100K |  |  |  |  |  |
| HPV 2011 Policy Dummy *×* 100K and Above | |  | 0·030 |  |  |
|  |  |  | (0·021) |  |  |
| HPV 2011 Policy Dummy *×* Undefined |  |  | -0·040* |  |  |
|  |  |  | (0·023) |  |  |
| By Education: Base Group: Grade 0-5 |  |  |  |  |  |
| HPV 2011 Policy Dummy *×* Grade 6-8 |  |  |  | -0·122 |  |
|  |  |  |  | (0·107) |  |
| HPV 2011 Policy Dummy *×* Grade 9-High School | |  |  | -0·042 |  |
|  |  |  |  | (0·104) |  |
| HPV 2011 Policy Dummy *×* Some College |  |  |  | -0·025 |  |
|  |  |  |  | (0·104) |  |
| HPV 2011 Policy Dummy *×* College and Above | |  |  | -0·132 |  |
|  |  |  |  | (0·104) |  |
| By Self-Reported Health Status: Base Group: Excellent/Very Good |  |  |  |  |  |
| HPV 2011 Policy Dummy *×* Good/Fair |  |  |  |  | 0·017 |
|  |  |  |  |  | (0·015) |
| HPV 2011 Policy Dummy *×* Poor |  |  |  |  | -0·174** |
|  |  |  |  |  | (0·070) |
| HPV 2011 Policy Dummy *×* Undefined |  |  |  |  | -0·082*** |
|  |  |  |  |  | (0·016) |
|  |  |  |  |  |  |
| Self-Reported Health Status |  |  |  |  |  |
| Good/Fair | 0·009 | 0·009 | 0·009 | 0·009 | 0·002 |
|  | (0·007) | (0·007) | (0·007) | (0·007) | (0·006) |
| Poor | -0·019 | -0·018 | -0·018 | -0·016 | 0·045 |
|  | (0·048) | (0·047) | (0·048) | (0·048) | (0·069) |
| Undefined | -0·050 | -0·049* | -0·049 | -0·054* | -0·008 |
|  | (0·033) | (0·029) | (0·030) | (0·030) | (0·012) |
| Education |  |  |  |  |  |
| Grade 6-8 | -0·008 | -0·009 | -0·008 | -0·003 | -0·008 |
|  | (0·014) | (0·014) | (0·014) | (0·013) | (0·014) |
| Grade 9-High School | -0·021 | -0·022 | -0·021 | -0·027* | -0·022 |
|  | (0·018) | (0·018) | (0·018) | (0·014) | (0·018) |
| Some College | 0·019 | 0·017 | 0·018 | 0·000 | 0·018 |
|  | (0·020) | (0·020) | (0·019) | (0·016) | (0·020) |
| College and Above | -0·046** | -0·046** | -0·049*** | -0·020 | -0·046** |
|  | (0·018) | (0·018) | (0·018) | (0·014) | (0·019) |
| Family Income |  |  |  |  |  |
| 100K and Above | 0·004 | 0·003 | -0·007 | 0·003 | 0·003 |
|  | (0·009) | (0·009) | (0·007) | (0·009) | (0·009) |
| Undefined | -0·030*** | -0·030*** | -0·013* | -0·030*** | -0·030*** |
|  | (0·010) | (0·010) | (0·008) | (0·010) | (0·010) |
| Race |  |  |  |  |  |
| Black | 0·006 | -0·008 | -0·009 | -0·009 | -0·008 |
|  | (0·007) | (0·008) | (0·008) | (0·008) | (0·008) |
| Other Races | -0·012* | -0·017* | -0·017* | -0·017* | -0·016* |
|  | (0·007) | (0·010) | (0·010) | (0·010) | (0·010) |
| Hispanic |  |  |  |  |  |
|  | 0·002 | 0·006 | 0·002 | 0·002 | 0·002 |
|  | (0·007) | (0·006) | (0·007) | (0·007) | (0·007) |
| Region |  |  |  |  |  |
| North Central/Midwest | -0·028*** | -0·029*** | -0·029*** | -0·029*** | -0·029*** |
|  | (0·010) | (0·010) | (0·010) | (0·010) | (0·010) |
| South | -0·036*** | -0·036*** | -0·036*** | -0·036*** | -0·036*** |
|  | (0·009) | (0·009) | (0·009) | (0·009) | (0·009) |
| West | -0·019* | -0·019* | -0·020* | -0·019* | -0·019* |
|  | (0·010) | (0·010) | (0·010) | (0·010) | (0·010) |
|  |  |  |  |  |  |
|  |  |  |  |  |  |
| Constant | 0·036*** | 0·039*** | 0·042*** | 0·040*** | 0·042*** |
|  | (0·011) | (0·011) | (0·011) | (0·011) | (0·011) |
|  |  |  |  |  |  |
|  |  |  |  |  |  |
| Age FE | Yes | Yes | Yes | Yes | Yes |
|  |  |  |  |  |  |
|  |  |  |  |  |  |
| No of Observations | 7,000 | 7,000 | 7,000 | 7,000 | 7,000 |
| Notes: FE is fixed effect. Robust standard errors are shown in parentheses. *** p<0·01, ** p<0·05, * p<0·1. The default (base-group) for Health Status is Excellent/Very good, for Education is Grade 0-5, Family Income is Below $100K, for Race is White, and for Region is Northeast. | | | | | |

| **Table S6: (Panel A) Spillover effects of the 2011 CDC recommendation policy for men aged 22-25, 26-40, and 41-60, without region by time trend.** | | | | | | |
| --- | --- | --- | --- | --- | --- | --- |
|  | Dependent Variable: Vaccination Status for Male Age Group 22-25 | | | | | |
|  | (1) | (2) | (3) | (4) | (5) | (6) |
| HPV 2011 Policy | 0·045*** | 0·044*** | 0·044*** | 0·044*** | 0·044*** | 0·044*** |
| Dummy | (0·005) | (0·005) | (0·005) | (0·005) | (0·005) | (0·005) |
|  |  |  |  |  |  |  |
| Race |  |  |  |  |  |  |
| Black |  |  | 0·003 | 0·003 | 0·007 | 0·006 |
|  |  |  | (0·008) | (0·008) | (0·008) | (0·008) |
| Other Races |  |  | -0·001 | -0·001 | -0·005 | -0·005 |
|  |  |  | (0·009) | (0·009) | (0·009) | (0·009) |
| Hispanic |  |  |  |  |  |  |
|  |  |  | -0·009 | -0·009 | -0·002 | -0·002 |
|  |  |  | (0·007) | (0·007) | (0·007) | (0·007) |
| Self-Reported Health Status |  |  |  |  |  |  |
| Good/Fair |  |  |  | -0·001 | 0·003 | 0·003 |
|  |  |  |  | (0·006) | (0·006) | (0·006) |
| Poor |  |  |  | 0·007 | 0·017 | 0·016 |
|  |  |  |  | (0·031) | (0·031) | (0·031) |
| Undefined |  |  |  |  |  |  |
|  |  |  |  |  |  |  |
| Education |  |  |  |  |  |  |
| Grade 6-8 |  |  |  |  | 0·049** | 0·049** |
|  |  |  |  |  | (0·021) | (0·021) |
| Grade 9-High School |  |  |  |  | 0·023*** | 0·024*** |
|  |  |  |  |  | (0·006) | (0·006) |
| Some College |  |  |  |  | 0·048*** | 0·049*** |
|  |  |  |  |  | (0·007) | (0·007) |
| College and Above |  |  |  |  | 0·064*** | 0·065*** |
|  |  |  |  |  | (0·019) | (0·019) |
| Family Income |  |  |  |  |  |  |
| 100K and Above |  |  |  |  |  | -0·013 |
|  |  |  |  |  |  | (0·010) |
| Undefined |  |  |  |  |  | -0·019* |
|  |  |  |  |  |  | (0·011) |
| Region |  |  |  |  |  |  |
| North Central/Midwest |  | -0·018* | -0·019* | -0·019* | -0·019* | -0·020* |
|  |  | (0·010) | (0·010) | (0·010) | (0·010) | (0·010) |
| South |  | -0·018* | -0·018* | -0·018* | -0·017* | -0·018* |
|  |  | (0·009) | (0·009) | (0·009) | (0·009) | (0·009) |
| West |  | -0·014 | -0·012 | -0·012 | -0·012 | -0·012 |
|  |  | (0·010) | (0·010) | (0·010) | (0·010) | (0·010) |
|  |  |  |  |  |  |  |
|  |  |  |  |  |  |  |
| Constant | 0·016*** | 0·030*** | 0·032*** | 0·032*** | -0·009 | -0·008 |
|  | (0·006) | (0·010) | (0·011) | (0·011) | (0·012) | (0·012) |
|  |  |  |  |  |  |  |
|  |  |  |  |  |  |  |
| Age FEs | No | Yes | Yes | Yes | Yes | Yes |
|  |  |  |  |  |  |  |
|  |  |  |  |  |  |  |
| No of Observations | 5,913 | 5,913 | 5,913 | 5,913 | 5,913 | 5,913 |
| Notes: FE is fixed effect. Robust standard errors are shown in parentheses. *** p<0·01, ** p<0·05, * p<0·1. The default (base-group) for Health Status is Excellent/Very good, for Education is Grade 0-5, Family Income is Below $100K, for Race is White, and for Region is Northeast. | | | | | | |

| **Table S6: (Panel B) Spillover effects of the 2011 CDC recommendation policy for men aged 22-25, 26-40, and 41-60, without region by time trend.** | | | | | | | | | | | | | | |
| --- | --- | --- | --- | --- | --- | --- | --- | --- | --- | --- | --- | --- | --- | --- |
|  | | Dependent Variable: Vaccination Status for Male Age Group 26-40 | | | | | | | | | | | | |
|  | | (1) | | (2) | | (3) | | (4) | | (5) | | | (6) | |
| HPV 2011 Policy | | 0·013*** | | 0·013*** | | 0·013*** | | 0·013*** | | 0·013*** | | | 0·013*** | |
| Dummy | | (0·001) | | (0·001) | | (0·001) | | (0·001) | | (0·001) | | | (0·001) | |
|  | |  | |  | |  | |  | |  | | |  | |
| Race | |  | |  | |  | |  | |  | | |  | |
| Black | |  | |  | | 0·009*** | | 0·008*** | | 0·009*** | | | 0·009*** | |
|  | |  | |  | | (0·003) | | (0·003) | | (0·003) | | | (0·003) | |
| Other Races | |  | |  | | 0·000 | | 0·000 | | -0·000 | | | -0·000 | |
|  | |  | |  | | (0·003) | | (0·003) | | (0·003) | | | (0·003) | |
| Hispanic | |  | |  | |  | |  | |  | | |  | |
|  | |  | |  | | -0·004** | | -0·004** | | -0·001 | | | -0·001 | |
|  | |  | |  | | (0·002) | | (0·002) | | (0·002) | | | (0·002) | |
| Self-Reported Health Status | |  | |  | |  | |  | |  | | |  | |
| Good/Fair | |  | |  | |  | | 0·003* | | 0·004** | | | 0·004** | |
|  | |  | |  | |  | | (0·002) | | (0·002) | | | (0·002) | |
| Poor | |  | |  | |  | | 0·030** | | 0·032** | | | 0·033** | |
|  | |  | |  | |  | | (0·014) | | (0·014) | | | (0·014) | |
| Undefined | |  | |  | |  | | -0·006 | | -0·006 | | | -0·006 | |
|  | |  | |  | |  | | (0·005) | | (0·005) | | | (0·006) | |
| Education | |  | |  | |  | |  | |  | | |  | |
| Grade 6-8 | |  | |  | |  | |  | | 0·002 | | | 0·002 | |
|  | |  | |  | |  | |  | | (0·005) | | | (0·005) | |
| Grade 9-High School | | | |  | |  | |  | | 0·001 | | | 0·001 | |
|  | |  | |  | |  | |  | | (0·004) | | | (0·004) | |
| Some College | | | |  | |  | |  | | 0·009* | | | 0·009* | |
|  | |  | |  | |  | |  | | (0·005) | | | (0·005) | |
| College and Above | | | |  | |  | |  | | 0·008 | | | 0·007 | |
|  | |  | |  | |  | |  | | (0·005) | | | (0·005) | |
| Family Income | |  | |  | |  | |  | |  | | |  | |
| 100K and Above | | | |  | |  | |  | |  | | | 0·002 | |
|  | |  | |  | |  | |  | |  | | | (0·002) | |
| Undefined | |  | |  | |  | |  | |  | | | 0·000 | |
|  | |  | |  | |  | |  | |  | | | (0·004) | |
| Region | |  | |  | |  | |  | |  | | |  | |
| North Central/Midwest | | | | -0·006** | | -0·006** | | -0·007*** | | -0·006** | | | -0·006** | |
|  | |  | | (0·003) | | (0·003) | | (0·003) | | (0·003) | | | (0·003) | |
| South | |  | | -0·004* | | -0·005** | | -0·005** | | -0·005** | | | -0·005** | |
|  | |  | | (0·002) | | (0·002) | | (0·002) | | (0·002) | | | (0·002) | |
| West | |  | | 0·002 | | 0·003 | | 0·003 | | 0·003 | | | 0·003 | |
|  | |  | | (0·003) | | (0·003) | | (0·003) | | (0·003) | | | (0·003) | |
|  | |  | |  | |  | |  | |  | | |  | |
|  | |  | |  | |  | |  | |  | | |  | |
| Constant | | 0·014*** | | 0·017*** | | 0·016*** | | 0·016*** | | 0·009 | | | 0·009 | |
|  | | (0·004) | | (0·004) | | (0·004) | | (0·004) | | (0·006) | | | (0·006) | |
|  | |  | |  | |  | |  | |  | | |  | |
|  | |  | |  | |  | |  | |  | | |  | |
| Age FEs | | No | | Yes | | Yes | | Yes | | Yes | | | Yes | |
|  | |  | |  | |  | |  | |  | | |  | |
|  | |  | |  | |  | |  | |  | | |  | |
| No of Observations | | 23,490 | | 23,490 | | 23,490 | | 23,490 | | 23,490 | | | 23,490 | |
| Notes: FE is fixed effect. Robust standard errors are shown in parentheses. *** p<0·01, ** p<0·05, * p<0·1. The default (base-group) for Health Status is Excellent/Very good, for Education is Grade 0-5, Family Income is Below $100K, for Race is White, and for Region is Northeast. | | | | | | | | | | | | | | |
| **Table S6: (Panel C) Spillover effects of the 2011 CDC recommendation policy for men aged 22-25, 26-40, and 41-60, without region by time trend.** | | | | | | | | | | | | | | |
|  |  | | Dependent Variable: Vaccination Status for Male Age Group 41-60 | | | | | | | | | | |  |
|  |  | | (1) | | (2) | | (3) | | (4) | | (5) | (6) | |  |
|  | HPV 2011 Policy | | 0·004*** | | 0·009*** | | 0·009*** | | 0·009*** | | 0·009*** | 0·009*** | |  |
|  | Dummy | | (0·001) | | (0·001) | | (0·001) | | (0·001) | | (0·001) | (0·001) | |  |
|  |  | |  | |  | |  | |  | |  |  | |  |
|  | Race | |  | |  | |  | |  | |  |  | |  |
|  | Black | |  | |  | | 0·002 | | 0·002 | | 0·002* | 0·002* | |  |
|  |  | |  | |  | | (0·001) | | (0·001) | | (0·001) | (0·001) | |  |
|  | Other Races | |  | |  | | 0·001 | | 0·001 | | 0·001 | 0·001 | |  |
|  |  | |  | |  | | (0·002) | | (0·002) | | (0·002) | (0·002) | |  |
|  | Hispanic | |  | |  | |  | |  | |  |  | |  |
|  |  | |  | |  | | -0·001 | | -0·001 | | -0·000 | -0·000 | |  |
|  |  | |  | |  | | (0·001) | | (0·001) | | (0·001) | (0·001) | |  |
|  | Self-Reported Health Status | |  | |  | |  | |  | |  |  | |  |
|  | Good/Fair | |  | |  | |  | | 0·000 | | 0·001 | 0·001 | |  |
|  |  | |  | |  | |  | | (0·001) | | (0·001) | (0·001) | |  |
|  | Poor | |  | |  | |  | | 0·001 | | 0·002 | 0·002 | |  |
|  |  | |  | |  | |  | | (0·002) | | (0·002) | (0·002) | |  |
|  | Undefined | |  | |  | |  | | -0·005*** | | -0·005*** | -0·005*** | |  |
|  |  | |  | |  | |  | | (0·001) | | (0·001) | (0·002) | |  |
|  | Education | |  | |  | |  | |  | |  |  | |  |
|  | Grade 6-8 | |  | |  | |  | |  | | 0·002 | 0·002 | |  |
|  |  | |  | |  | |  | |  | | (0·001) | (0·001) | |  |
|  | Grade 9-High School | | | |  | |  | |  | | 0·003*** | 0·003*** | |  |
|  |  | |  | |  | |  | |  | | (0·001) | (0·001) | |  |
|  | Some College | | | |  | |  | |  | | 0·006*** | 0·007*** | |  |
|  |  | |  | |  | |  | |  | | (0·001) | (0·001) | |  |
|  | College and Above | | | |  | |  | |  | | 0·006*** | 0·007*** | |  |
|  |  | |  | |  | |  | |  | | (0·002) | (0·002) | |  |
|  | Family Income | |  | |  | |  | |  | |  |  | |  |
|  | 100K and Above | | | |  | |  | |  | |  | -0·001 | |  |
|  |  | |  | |  | |  | |  | |  | (0·001) | |  |
|  | Undefined | |  | |  | |  | |  | |  | -0·003** | |  |
|  |  | |  | |  | |  | |  | |  | (0·001) | |  |
|  | Region | |  | |  | |  | |  | |  |  | |  |
|  | North Central/Midwest | | | | 0·000 | | 0·000 | | 0·000 | | 0·000 | -0·000 | |  |
|  |  | |  | | (0·001) | | (0·001) | | (0·001) | | (0·001) | (0·001) | |  |
|  | South | |  | | 0·002* | | 0·002 | | 0·002 | | 0·002 | 0·002 | |  |
|  |  | |  | | (0·001) | | (0·001) | | (0·001) | | (0·001) | (0·001) | |  |
|  | West | |  | | 0·003** | | 0·003** | | 0·003** | | 0·003** | 0·003** | |  |
|  |  | |  | | (0·001) | | (0·001) | | (0·001) | | (0·001) | (0·001) | |  |
|  |  | |  | |  | |  | |  | |  |  | |  |
|  |  | |  | |  | |  | |  | |  |  | |  |
|  | Constant | | 0·002 | | 0·000 | | 0·000 | | 0·000 | | -0·005** | -0·005** | |  |
|  |  | | (0·002) | | (0·002) | | (0·002) | | (0·002) | | (0·002) | (0·002) | |  |
|  |  | |  | |  | |  | |  | |  |  | |  |
|  |  | |  | |  | |  | |  | |  |  | |  |
|  |  | |  | |  | |  | |  | |  |  | |  |
|  | Age FEs | | No | | Yes | | Yes | | Yes | | Yes | Yes | |  |
|  |  | |  | |  | |  | |  | |  |  | |  |
|  |  | |  | |  | |  | |  | |  |  | |  |
|  | No of Observations | | 31,284 | | 31,284 | | 31,284 | | 31,284 | | 31,284 | 31,284 | |  |
|  | Notes: FE is fixed effect. Robust standard errors are shown in parentheses. *** p<0·01, ** p<0·05, * p<0·1. The default (base-group) for Health Status is Excellent/Very good, for Education is Grade 0-5, Family Income is Below $100K, for Race is White, and for Region is Northeast. | | | | | | | | | | | | |  |
